# Frykman Type 7-8 Distal Radius Fractures in Elderly Patients: Conservative Treatment vs Volar Plating

**DOI:** 10.7759/cureus.63035

**Published:** 2024-06-24

**Authors:** Mehmed Nuri Tutuncu, Murat Demiroğlu

**Affiliations:** 1 Orthopaedics and Traumatology, Göztepe Prof. Dr. Süleyman Yalçın City Hospital, Istanbul, TUR; 2 Orthopaedics and Traumatology, Ataşehir Florence Nightingale Hospital, Istanbul, TUR

**Keywords:** casting, frykman classification, plate fixation, distal radius fracture, elderly

## Abstract

Introduction: The aim of this study was to determine the clinical outcomes of conservative and surgical treatments in elderly patients with displaced Frykman type 7-8 distal radius fractures.

Methods: The clinical outcomes of 50 patients aged 60 and older with displaced Frykman type 7-8 fractures who underwent surgical and conservative treatments between January 2019 and January 2022 were determined. The joint range of motion, pain scores, functional scores, radiological parameters, and any complications that occurred posttreatment were evaluated for each patient who underwent both treatments.

Results: Descriptive characteristics, excluding sex, were evaluated in 18 patients treated with casting and 32 patients treated with volar plating, and no statistically significant differences were detected between the groups. The functional and radiological assessments of the groups showed no significant differences (p>0.05). The volar tilt of patients who underwent surgical treatment was significantly greater than that of patients who were treated with a cast (p=0.02). The Mayo wrist scores of patients with step-offs greater than 2 mm were significantly lower (p=0.007; p<0.01). The visual analog scale (VAS) scores of patients who met the step-off criterion were significantly greater (p=0.025; p<0.05). The Mayo wrist scores of patients whose radiological parameters were within acceptable limits were significantly greater (p=0.007; p<0.01). The Quick-Quick Disabilities of the Arm, Shoulder, and Hand (DASH) scores of patients whose radiological parameters were within acceptable limits were significantly lower (p=0.007; p<0.01).

Conclusion: In elderly patients with identified Frykman type 7-8 fractures, casting and volar plating treatments produced similar results. Especially in patients with low expectations and multiple comorbidities, satisfactory results can be achieved with plaster treatment.

## Introduction

Distal radius fractures are common injuries encountered in emergency orthopedic practice. The frequency of these fractures is increasing, and in some series, they generally constitute the first or second most common type of fracture [1,2]. Osteoporosis is detectable in the elderly population, while high-energy trauma is identified as the etiology in the younger age group [3].

The Frykman classification of distal radius fractures provides an idea about the degree of fragmentation of the fracture. In brief, the Frykman classification categorizes fractures of the distal end of the radius into types one through eight. Types 1-2 represent extra-articular fractures, types 3-4 are fractures that reach only the radiocarpal joint, types 5-6 reach only the radioulnar joint, and types 7-8 reach both the radioulnar and radiocarpal joints. Even numbers indicate that an ulnar styloid fracture was added to the respective fracture type [4]. Type 7-8 fractures are the most severely fragmented type, often requiring surgical treatment for management [5]. In these types of fractures, even if acceptable radiological parameters are achieved after closed reduction, reduction losses of up to 64% can occur [6].

Lafontaine et al. reported instability criteria that could lead to reduction loss in fractures of the distal end of the radius. These criteria include, in sequence, the extension of the fracture to the radiocarpal joint, age 60 and older, dorsal fragmentation, dorsal angulation exceeding 20 degrees at the initial presentation, and the presence of an ulnar styloid fracture. If three of these criteria are met, the fracture is defined as unstable, and it is emphasized that there is a strong possibility of reduction loss [7].

In clinical practice, a portion of elderly individuals may refuse surgical treatment following a wrist fracture. The potential limitation of movement in the wrist is accepted against the possible risks of surgical treatment. In the current institution, surgical treatment is recommended for fractures of this type that are highly fragmented, regardless of age group. Since the patients included in this study were aged 60 years and older and had displaced Frykman type 7-8 fractures, it was generally assumed that their fractures were unstable. Therefore, surgical treatment was recommended, even if the post-reduction position was deemed acceptable, as reduction loss could occur. Some authors have suggested that conservative treatment may be sufficient even in elderly patients with fragmented unstable fractures, as functional outcomes with fracture malreduction may not necessarily be worse in these patients than in younger patients [8,9].

This study presents a comparison of the functional outcomes of patients who refused surgical treatment for these types of unstable fragmented fractures with those of patients who underwent surgical treatment.

## Materials and methods

In this study, 50 patients aged 60 years and older with displaced Frykman type 7-8 fractures accompanied by initial dorsal angulation exceeding 20 degrees between 2019 and 2022 were retrospectively screened and included. Local ethics committee approval was obtained from international agreements (Helsinki Declaration), and written consent was obtained from the patients (date: 21/09/2022, number: 0543).

Despite radiological acceptability within admissible limits after reduction in patients with this type of fracture, surgical treatment was recommended for all patients. Some patients refused surgical treatment. The pain scores, radiological parameters, and functional outcomes of 32 patients who underwent surgical treatment and 18 patients who received conservative treatment were analyzed.

A significant portion of patients who refused surgical treatment were individuals who avoided surgery and hospitalization during the period coinciding with the COVID-19 pandemic. An upper extremity surgeon treated all patients with 10 years of experience in this field (MD).

The inclusion criterion was patients aged 60 years and older with a follow-up of at least two years. Patients presented with a Frykman type 7-8 fracture with initial dorsal angulation exceeding 20 degrees. Patients who accepted surgical treatment were included in the surgery group, while those who refused surgical treatment were included in the conservative treatment group.

The exclusion criteria included open fractures, nondisplaced fractures, a history of fractures in the same extremity, rheumatologic diseases, neurovascular deficits, a history of malignancy, steroid use, and peripheral arterial disease.

Patient demographic data, including age, sex, side, smoking habits, and dominant side of the upper extremity, were recorded. After routine permission to return to work, patients were called for follow-ups in the third month, sixth month, first year, and subsequent years. Routine anteroposterior and lateral radiographs were taken. In the second year of follow-up, patients were compared in terms of the Quick Disabilities of the Arm, Shoulder, and Hand (DASH) score [10] for quality of life related to the upper extremity, Mayo wrist score, radiological parameters (radial inclination, step off, and volar tilt), visual analog scale (VAS, 0 indicating no pain and 10 indicating worst pain) assessment, joint range of motion, and complications [10].

Radiological angle measurements were performed by two researchers using digital software on the radiographs, and the average values were recorded. Patients with a step of more than 2 mm were considered to have stepped off. The overall alignment of the distal radius fracture was considered "unacceptable" if the dorsal angulation was 10° or if the radial inclination was less than 15° according to the American Society for Surgery of the Hand (ASSH) guidelines [11].

Joint movements were measured using a goniometer. At the initial presentation, routine anteroposterior and lateral radiographs were taken. Computerized tomography (CT) scans were performed for patients suspected of having joint extension. Subsequently, fracture types were classified according to the Frykman classification, and patients aged 60 years and older with type 7-8 fractures were included in the study.

Conservative treatment protocol

Nonsurgical treatment is based on using a full forearm cast for complete fractures. The cast is shaped with the wrist in mild flexion and ulnar deviation for the carpus. All patients were checked every 10 days, and an X-ray was taken. The cast was removed at six weeks. After the removal of the cast, patients were instructed to perform a joint range of motion exercises and were scheduled for a follow-up appointment one week later. Patients whose joint range of motion did not approach normal values were referred to physiotherapy. Return-to-work permission was granted after the eighth week, while those engaged in heavy labor were allowed to return in the 10th week.

Surgical treatment protocol

Postreduction, fixation was applied using a standard locking compression plate and screws through a modified volar Henry incision. All surgeries were performed under a tourniquet, under general anesthesia, and with the assistance of fluoroscopy. A light splint was applied for three weeks, and during the immobilization period, active and passive finger and elbow exercises were advised. From the third week onward, the patients were taught to perform joint range of motion exercises, and those with limitations in the range of motion of the joints after one week were referred to physiotherapy.

Statistical analysis

The findings of the study were evaluated using Statistical Product and Service Solutions (SPSS, version 26.0.; IBM SPSS Statistics for Windows, Armonk, NY) for the statistical analyses. When assessing the study data, descriptive statistical methods such as the mean, standard deviation, median, minimum, and maximum values were used for quantitative variables, and frequencies and percentages were used for qualitative variables.

The Shapiro-Wilks test and box plot graphs were used to assess the normality of the distribution of the data. For quantitative variables showing a normal distribution between two groups, Student’s t-test was used. For variables that did not exhibit a normal distribution, the Mann-Whitney U test was used for comparisons between the two groups. The chi-square test and Fisher’s exact test were used for the comparison of qualitative data. The results were evaluated at a significance level of p < 0.05 with a 95% confidence interval.

## Results

This study included a total of 50 patients, with 60% (n=30) being female and 40% (n=20) being male. The ages of the patients ranged from 60 to 85 years, with a mean of 68.24±7.74 years. Among the patients, 36% (n=18) were treated with a cast, while 64% (n=32) underwent surgery. Among the patients included in the study, 64% (n=32) had fractures on their dominant side, while 36% (n=18) had fractures on their nondominant side. Fractures were more common in the dominant upper extremity. Complications of complex regional pain syndrome were observed in 6% of the patients (n=3), all of whom were in the surgical group. Seventy-two percent of the patients included in the study (n=36) had radiological parameters within acceptable limits (Table 1).

**Table 1:** Distribution of Descriptive Characteristics n: Number, SD: Standart Deviation, Min: Minimum, Max: Maximum

		n (%)
Gender	Female	30 (60.0)
	Male	20 (40.0)
Age	Mean±SD	68.24±7.74
	Median (Min-Max)	64.5 (60-85)
Comorbidity	Absent	24 (48.0)
	Present	26 (52.0)
Cigarette (package/year)	Absent	32 (64.0)
	Present	18 (36.0)
	Mean±SD	32.22±12.03
	Median (Min-Max)	30 (5-50)
Treatment	Casting	18 (36.0)
	Plating	32 (64.0)
Side	Right	18 (36.0)
	Left	32 (64.0)
Fractured side	Dominant	32 (64.0)
	Non-dominant	18 (36.0)
Complication	Absent	47 (94.0)
	Present	3 (6.0)
	Complex regional pain syndrome	3
Mechanism of injury	Fall	46 (92.0)
	Traffic accident	1 (2.0)
	Fall from high	3 (6.0)
Radiological parameters	Acceptable	36 (72.0)
	Not acceptable	14 (28.0)
Step off	Absent	11 (22.0)
	Present	39 (78.0)

The rate of surgical treatment in male patients was significantly greater than that in female patients (p=0.002; p<0.01). Age, smoking habits, fracture site, and the presence of complications were not significantly different between the groups (p>0.05). The rate of plaster treatment in patients with comorbidities was substantially greater than that of surgical treatment (p=0.032; p<0.05) (Table 2).

**Table 2:** Comparison of Descriptive Characteristics According to Treatment Types n: Number, SD: Standart Deviation, Min: Minimum, Max: Maximum ^a^Pearson chi-square test, ^b^Student-T test, **p<0.01, *p<0.05

		Tedavi		p
		Casting (n=18)	Plating (n=32)	
Gender	Female	16 (88.9)	14 (43.8)	^a^0.002**
	Male	2 (11.1)	18 (56.3)	
Age	Mean±SD	68.50±7.64	68.09±7.33	^b^0.272
	Median (Min-Max)	64 (60-85)	66 (60-83)	
Comorbidity	Absent	5 (27.8)	19 (59.4)	^a^0.032*
	Present	13 (72.2)	13 (40.6)	
Cigaratte	Absent	11 (61.1)	21 (65.6)	^a^0.750
	Present	7 (38.9)	11 (34.4)	
Fractured side	Dominant	13 (72.2)	19 (59.4)	^a^0.364
	Nondominat	5 (27.8)	13 (40.6)	
Complication	Absent	18 (100)	29 (90.6)	^a^0.180
	Present	0 (0)	3 (9.4)	

The measurements of flexion, extension, supination, and pronation did not significantly differ according to treatment type (p>0.05) (Table 3).

**Table 3:** Comparison of Joint Range of Motion According to Treatment Types n: Number, SD: Standart Deviation, Min: Minimum, Max: Maximum ^b^Student t-test, ^c^Mann-Whitney U test, *p<0.05

			Treatment		p
		Sum	Casting (n=18)	Plating (n=32)	
Flexion	Mean±SD	64.20±13.26	63.61±15.98	64.53±11.73	^c^0.594
	Median (Min-Max)	70 (30-80)	70 (30-80)	65 (40-80)	
Extension	Mean±SD	60.60±12.52	61.11±8.14	60.31±14.53	^b^0.831
	Median (Min-Max)	60 (30-80)	60 (40-80)	60 (30-80)	
Supination	Mean±SD	62.3±10.11	61.94±9.87	62.5±10.4	^c^0.732
	Median (Min-Max)	60 (40-80)	60 (40-80)	60 (40-80)	
Pronation	Mean±SD	63.5±11.03	61.94±9.87	64.38±11.69	^b^0.460
	Median (Min-Max)	65 (40-80)	62,5 (40-80)	65 (40-80)	

The Mayo wrist, Quick-DASH, and VAS scores did not significantly differ among the patients based on treatment type (p>0.05). Similarly, there was no statistically significant difference among groups regarding Frykman type, the presence of radiological step-off, being within acceptable limits, or inclination values (p>0.05). However, the tilt measurements for patients who underwent surgical treatment were significantly greater than those for patients who underwent plaster treatment (p=0.020; p<0.05) (Table 4).

**Table 4:** Comparison of Clinical Characteristics Based on Treatment Types n: Number, SD: Standart Deviation, Min: Minimum, Max: Maximum, VAS: Visual Analog Scale ^a^Pearson chi-square test, ^b^Student t-test, ^c^Mann-Whitney U test, ^d^Fischer's exact test, *p<0.05

			Treatment		p
		Sum	Casting (n=18)	Plating (n=32)	
Mayo wrist score	Mean±SD	81.5±10.16	81.11±10.65	81.72±10.05	^c^0.812
	Median (Min-Max)	85 (50-100)	77.5 (65-100)	85 (50-90)	
Quick-DASH	Mean±SD	6.29±4.64	7.77±4.71	5.47±4.46	^b^0.093
	Median (Min-Max)	5.9 (0-20)	6.8 (0-20)	4.5 (0-15.9)	
VAS	Mean±SD	0.92±1.03	0.89±0.96	0.94±1.08	^c^0.897
	Median (Min-Max)	1 (0-3)	1 (0-3)	1 (0-3)	
İnclination	Mean±SD	16.52±6.00	15.72±8.17	16.97±4.45	^b^0.485
	Median (Min-Max)	18 (0-27)	18.5 (0-27)	18 (4-22)	
Volar tilt	Mean±SD	5.43±10.02	1.08±14.04	7.88±5.80	^b^0.020*
	Median (Min-Max)	7 (-25/25)	0 (-25/25)	8.5 (-3/16)	
Step off	Absent	39 (78.0)	12 (66.7)	27 (84.4)	^d^0.172
	Present	11 (22.0)	6 (33.3)	5 (15.6)	
Frykman type	Type 7	28 (56.0)	8 (44.4)	20 (62.5)	^a^0.217
	Type 8	22 (44.0)	10 (55.6)	12 (37.5)	
Radiological parameters	Acceptable	36 (72.0)	12 (66.7)	24 (75.0)	^a^0.529
	Not acceptable	14 (28.0)	6 (33.3)	8 (25.0)	

The Mayo wrist scores of patients who stepped off were significantly lower (p=0.007; p<0.01). The VAS scores of patients who underwent step-off surgery were significantly greater (p=0.025; p<0.05). No statistically significant difference was observed in the Quick-DASH score among the patients based on the presence of a step-off (p>0.05). In patients who achieved step-off, functional outcomes deteriorated, accompanied by an increase in pain scores (Table 5).

**Table 5:** Comparison of Clinical Scores Based on the Presence of Step-Off n: Number, SD: Standart Deviation, Min: Minimum, Max: Maximum, VAS: Visual Analog Scale ^b^Student t-test, ^c^Mann-Whitney U test, *p<0.05, **p<0.01

		Step-off		p
		Absent	Present	
Mayo wrist score	Mean±SD	83.08±10.61	75.91±5.84	^c^0.007**
	Median (Min-Max)	85 (50-100)	75 (65-85)	
Quick DASH	Mean±SD	5.62±4.66	8.7±3.87	^b^0.051
	Median (Min-Max)	4.5 (0-20)	9.1 (4.5-13.6)	
VAS score	Mean±SD	0.74±0.94	1.55±1.13	^c^0.025*
	Median (Min-Max)	0 (0-3)	1 (0-3)	

Patients whose radiological parameters were within acceptable limits had significantly greater Mayo wrist scores (p=0.007; p<0.01). Similarly, the Quick-DASH scores of patients whose radiological parameters were within acceptable limits were significantly lower (p=0.007; p<0.01). No statistically significant difference was found in VAS scores among patients based on radiological parameter limits (p>0.05). Although the functional outcomes of patients whose radiologically acceptable limits improved, their pain scores did not significantly differ (Table 6).

**Table 6:** Comparison of Clinical Scores Based on Radiological Acceptability n: Number, SD: Standart Deviation, Min: Minimum, Max: Maximum, VAS: Visual Analog Scale ^b^Student t-test, ^c^Mann-Whitney U test, *p<0.05, **p<0.01

		Radiological parameters		p
		Acceptable	Not acceptable	
Mayo wrist score	Mean±SD	83.89±9.86	75.36±8.43	^c^0.002**
	Median (Min-Max)	85 (50-100)	75 (60-90)	
Quick DASH	Mean±SD	5.40±4.19	8.59±5.1	^b^0.027*
	Median (Min-Max)	4.5 (0-15.9)	6.8 (2.3-20)	
VAS	Mean±SD	0.83±1.03	1.14±1.03	^c^0.249
	Median (Min-Max)	0.5 (0-3)	1 (0-3)	

## Discussion

In the elderly population, distal radius fractures are among the most frequently encountered fractures, yet it is impossible to define the gold standard treatment method. In this study, no significant differences were found in the Mayo wrist or Quick-DASH scores, range of motion (ROM) values, or VAS scores based on treatment type. Patients who underwent articular step-off were observed to have significantly lower Mayo wrist scores, higher VAS scores, and no significant difference in Quick-DASH scores. Patients who did not fall within radiologically acceptable limits were found to have significantly lower Mayo scores, higher Quick-DASH scores, and no significant difference in VAS scores.

In the present study, no statistically significant differences were observed in the descriptive characteristics of the groups, except for the sex factor. A significantly greater proportion of females was found in the plaster group. This could be interpreted as females being more reluctant to undergo surgery. In addition, the groups are statistically homogeneous. Complications were observed in three patients (6%), all of whom were in the surgical group (complex regional pain syndrome), but the differences were not statistically significant. Arora et al. [12], in their prospective study, reported a greater frequency of complications in the surgical group. In a meta-analysis examining studies on open reduction internal fixation (ORIF) and closed reduction with casting in the elderly population, no significant difference in complications between the groups was found [13]. The most common complication reported was complex regional pain syndrome. The rate of conservative treatment in patients with additional diseases was significantly greater (p<0.05). It can be inferred that patients with more comorbidities tend to avoid the risks of surgery and, if necessary, general anesthesia.

No statistically significant differences were observed in the ROM values according to the treatment type. Yu et al., in their meta-analysis, reported that the posttreatment supination ROM was significantly greater in the conservative group, but other values did not significantly differ [13]. In a case-control study examining intra-articular fractures, Egol et al. [14] reported that, at the end of one year, supination movement was significantly greater in the conservative treatment group, while other movements were similar. Martinez-Mendez et al. [15], in a prospective randomized controlled trial investigating intra-articular distal radius fractures in the elderly population (plate/casting), emphasized that flexion and extension ranges were similar after two years, but supination and pronation values were better in the surgical group.

In the surgical group in the present study, numerically lower articular step-off, greater radiological acceptability, and greater radial inclination were observed, although these differences were not statistically significant. However, the volar tilt was significantly greater in the surgical group (p<0.05). Many studies have reported better radiological outcomes in surgical groups [12,14-16]. It is obvious that, when fractures are treated surgically, existing deformities can be better corrected. In this study, there were no significant differences between groups in terms of the Mayo wrist score, Quick-DASH score, or VAS score. Arora et al., in a retrospective comparative study conducted on patients over 70 years old, reported similar functional scores between groups at two years (casting/plating), with lower VAS scores in the plaster group [16]. Avcı et al. [17], in a retrospective study conducted on patients over 75 years old, demonstrated no difference in pain or functional scores between groups at one year (casting/plating). In a prospective randomized controlled study conducted on patients over 50 years old, Sirniö et al. [18] reported better functional scores in the surgical group than in the conservative treatment group at one year. Different study concepts, age groups, and patient populations in the literature have led to diverse results in various studies.

According to the analysis of the presence of step-offs (≥2 mm) in all patients who participated in the study, the Mayo wrist score was significantly lower (p<0.01), the VAS score was significantly greater (p<0.05), and the Quick-DASH score was not significantly different (p=0.51). Mendez et al. [15], in a prospective randomized study, reported that articular step-off did not affect functional outcomes. Hegeman et al. [19], in a study of patients over 55 years old with unstable intra-articular fractures treated with closed reduction and external fixation, reported that joint incongruity was consistent with poor functional outcomes.

In terms of radiological acceptability, all patients were evaluated for functional outcomes and pain scores. The Mayo wrist scores were significantly lower, and the Quick-DASH scores were significantly greater in patients who were outside the acceptable limits. However, VAS scores were not significantly different. In a study by Raudasoja et al. [20], which included patients with AO type C distal radius fractures in the general population, they compared fracture healing with malalignment and healing within acceptable radiological limits. They found that functional scores did not significantly differ after treatment. In this regard, there is no consensus in the literature [12,13,16,20].

Hustedt et al. [21], in a meta-regression analysis that included publications comparing conservative treatment and plating for distal radius fractures in elderly patients, reported that patients aged 60-70 years who underwent plate and screw fixation had significantly better functional outcomes. The effectiveness of this intervention decreased in the 70-80-year-old group and was similar in patients over 80 years old.

This study has several limitations. First, since it was designed retrospectively, the level of evidence is low. The sample sizes of the groups may be considered low. Forming the conservative treatment group from patients who refused surgery may create some biases and negatively affect randomization. The conservative patient group consisted of individuals with more comorbidities and likely lower functional expectations. This may lead to supportive findings for conservative treatment when comparing post-treatment functional outcomes.

Overall, personalized treatment planning is essential for patients with this type of fracture. Factors such as the patient's age, activity level, expectations about treatment, and expected outcomes of the chosen treatment should be thoroughly discussed with the patient to make an informed decision.

## Conclusions

Conservative treatment can yield good functional results in elderly patients, especially in patients whose articular step-off is less than 2 mm. Although not within radiologically acceptable limits, conservative treatment may still provide satisfactory results for less demanding patients. Consequently, individualized treatment planning remains essential to achieving favorable outcomes.
